# Ubiquitin‐Proteasome System–Related Prognostic Model and Immune Landscape in Glioblastoma

**DOI:** 10.1155/ijog/2045937

**Published:** 2026-07-22

**Authors:** Da Huo, Yue Feng, Zheng Yu, Wanting Xie, Hai Jin

**Affiliations:** ^1^ Department of Neurosurgery, General Hospital of Northern Theater Command, Shenyang, China, syjqzyy.com; ^2^ Department of General Practice, General Hospital of Northern Theater Command, Shenyang, China, syjqzyy.com; ^3^ Department of Laboratory, 32295 Army Hospital, Liaoyang, China; ^4^ Department of Nursing, General Hospital of Northern Theater Command, Shenyang, China, syjqzyy.com

**Keywords:** glioblastoma, immunity, macrophage, prognosis, scRNA-Seq, spatial transcriptome

## Abstract

**Background:**

Glioblastoma (GBM) is a highly aggressive brain tumor with poor prognosis. This study is aimed at establishing an ubiquitin‐proteasome system (UPS)–related prognostic model and investigating its link to immune infiltration and therapy response.

**Materials and Methods:**

GBM datasets were obtained from public databases. Ubiquitin‐proteasome system–related genes (UPSGs) were identified from literature. Consensus clustering defined UPS‐based GBM subtypes. Differentially expressed genes (DEGs) were screened, and a prognostic model was constructed using univariate Cox, least absolute shrinkage and selection operator (LASSO), and stepwise regression. The model′s performance was validated using survival analysis and time‐dependent receiver operating characteristic (ROC) curves. Immune infiltration was assessed using single‐sample gene set enrichment analysis (ssGSEA), TIMER, and ESTIMATE. Drug sensitivity was assessed by correlating the half‐maximal inhibitory concentration (IC_50_) of candidate drugs with the risk score. Single‐cell RNA sequencing data were used to characterize UPSG expression across distinct cell subpopulations in GBM. For in vitro validation, key UPSGs were silenced in GBM cell lines, and cell proliferation, migration, and invasion were measured using Cell Counting Kit‐8 (CCK‐8), wound healing, and Transwell assays, respectively.

**Results:**

Two UPS‐related GBM subtypes were identified. Six genes (*IGFBP6*, *CTSD*, *SPAG4*, *ZNF560*, *COL22A1*, and *HOXC13*) formed the prognostic model, where high Riskscore indicated poor survival. High Riskscore correlated with greater immune infiltration, including CD8^+^ T cells and macrophages. IC_50_ values of 24 drugs were significantly associated with Riskscore. Single‐cell analysis revealed seven GBM subpopulations; notably, *COL22A1* was enriched in MES‐like cells, and CTSD in macrophages. *IGFBP6* promoted GBM cell proliferation, migration, and invasion.

**Conclusion:**

This study establishes a UPS‐based prognostic model for GBM that links immune infiltration and drug sensitivity, providing potential biomarkers and therapeutic targets for GBM.

## 1. Introduction

Glioma is an intracranial malignant tumor with high aggressiveness and high morbidity and mortality [1, 2]. Glioma patients have no typical symptoms in the early stage, which makes it easy to miss the best time for treatment [3, 4]. Gliomas also have a very high degree of malignancy, which leads to poor surgical treatment and a high recurrence rate in a short period of time, which is a serious threat to patients′ life safety [5, 6]. Among them, glioblastoma (GBM) is the highest grade of glioma, and the relative 5‐year survival rate of patients is less than 5%, and the prognosis is not optimistic [7, 8]. Despite advances in both the molecular pathogenesis and the treatment of GBM over the past decade, overall patient survival remains poor, with a median survival of only 12–15 months [9–11]. Currently, molecular markers are included as part of the classification of brain tumors such as GBM, especially in the tumor classification criteria issued by the World Health Organization, where gliomas containing specific molecular mutations are classified separately, highlighting the importance of molecular biomarkers in the pathological analysis of GBM [12]. Thus, the search for reliable GBM biomarkers is crucial for the diagnosis and treatment of GBM.

Dynamic regulation of the ubiquitin‐proteasome system (UPS) is tightly linked to the development of malignant tumors [13, 14]. The UPS is essential for maintaining protein dynamic homeostasis and for regulating eukaryotic processes, including the nonlysosomal degradation of oxidized, damaged, or misfolded proteins in eukaryotic cells [15]. This process is tightly regulated by activation and transfer of ubiquitin chains to target proteins, which are subsequently recognized and degraded by the 26S proteasome complex [16]. The role of the UPS is to regulate protein levels through degradation in order to maintain essential cellular processes such as growth, division, and signaling, whereas dysregulation of the UPS leads to a loss of proteolysis and of the ability to maintain protein mass, which is closely associated with various malignant tumor development is closely linked to the development of various malignant tumors [17]. Aberrant expression of oncogenes and oncogene products is prevalent in gliomas, and UPS can influence their development through the regulation of various pathways in glioma cells [18]. For example, the E3 ubiquitin ligases MDM2 and RING1 in many types of cancer cells, including glioma cells, interact with the oncogene p53 and are able to enhance the p53 degradation process, and ubiquitination modifications degrade the tumor suppressor in these segments to achieve the role of promoting cancer cell growth [19, 20]. In addition, certain E3 ubiquitinating enzymes can activate the PI3K‐AKT signaling pathway and promote cancer cell metastasis, which has implications for the elucidation of mechanisms related to glioma cell invasion and migration [21, 22].

This study presents a novel integration of the UPS with GBM prognosis and immune biology. Unlike previous work focusing on individual UPS genes, we systematically characterized UPS‐related molecular subtypes and built a prognostic signature that links tumor aggressiveness to immune infiltration and drug response. By leveraging bulk, single‐cell, and spatial transcriptomic data, our approach reveals cell‐type–specific expression patterns of UPS genes within the tumor microenvironment. The findings offer not only a practical risk stratification tool but also potential targets for modulating immune evasion and overcoming chemoresistance in GBM.

## 2. Materials and Methods

### 2.1. Data Acquisition

In this study, TPM data were downloaded from the UCSC xena database (https://gdc-hub.s3.us-east-1.amazonaws.com/download/TCGA-GBM.star_tpm.tsv.gz) of The Cancer Genome Atlas (TCGA) dataset for the GBM dataset, which was screened to contain a total of 145 tumor samples. The mRNAseq_325 dataset was downloaded from the Chinese Glioma Genome Atlas (CGGA, http://www.cgga.org.cn/) database containing 135 GBM samples. The single‐cell dataset GSE273274 for GBM was downloaded from the Gene Expression Omnibus (GEO, https://www.ncbi.nlm.nih.gov/geo) database, including three center samples of GBM. The spatial transcriptome dataset GSE273275 of GBM was downloaded from the GEO database, including the center sample of one case of GBM. The genes associated with the UPS used in this study were obtained from the relevant literature [23].

### 2.2. Molecular Subtype Identification Based on UPSGs

In this study, the prognostic relevance of UPSGs was clarified by univariate Cox regression analysis, and the *p* value of less than 0.05 was used as a criterion to screen the prognostically significant UPSGs and used to identify the molecular subtypes of GBM. Subsequently, a consistency matrix was constructed using consistency clustering [24] to identify molecular subtypes of GBM based on the expression profiles of UPSGs significantly associated with prognosis. In this study, the km algorithm and 1‐Spearman correlation were used as the metric distances, and the clustering stability was validated through bootstrap resampling—a statistical method that involves random sampling with replacement to estimate the robustness of results. Specifically, 1000 bootstraps were performed, with each bootstrap process including 80% of the training set of patients and a set number of clusters from 2 to 10.

### 2.3. Differential Expressed Analysis and Functional Analysis

In this study, UPS‐associated differentially expressed genes (DEGs) among subtypes of GBM samples were analyzed using the R package limma [25], with *p* value of < 0.05 and |log_2_ *F* *C*| > log_2_(1.5) as the screening criteria. This study was followed up by Gene Ontology (GO) and Kyoto Encyclopedia of Genes and Genomes (KEGG) functional enrichment analysis of these UPS‐associated DEGs by R package clusterProfiler.

### 2.4. GBM Prognostic Risk Assessment Model Construction and Validation

In this study, the UPS‐related DEGs were analyzed by univariate Cox regression, and the *p* value less than 0.01 was used as the screening criterion. Then, LASSO regression using the R package glmnet was performed to further compress these genes, and the number of genes was narrowed down by stepwise regression analysis. This was used to construct a GBM risk assessment model, and the formula for model construction is shown below.

Riskscore=∑βi∗expression i

where *i* refers to the gene expression level, and *β* is the Cox regression coefficient of the corresponding gene.

Next, *z* score processing on Riskscore was implemented based on the threshold “0” to divide the GBM sample (TCGA‐GBM and mRNAseq_325) into the high‐ and low‐Riskscore groups and Kaplan–Meier survival analysis was performed. Subsequently, the receiver operating characteristic curve (ROC) was plotted [26]. To explore the functional differences between the high‐ and low‐Riskscore groups, a GSEA analysis between the subgroups was additionally performed.

### 2.5. Analysis of Immune Infiltration Levels

In this study, the immune components of the tumor microenvironment of TCGA‐GBM were quantified by the ESTIMATE algorithm, which led to the ImmuneScore for assessing the level of immune cell infiltration. The enrichment score of 28 immune cells in each GBM sample was calculated using ssGSEA [27]. Immune cell infiltration in different GBM subgroups was assessed using the TIMER algorithm.

### 2.6. Correlation Analysis of Riskscore With GBM Drug Sensitivity

In this study, R package oncoPredict was used for prediction of IC_50_ values on TCGA‐GBM dataset. Spearman correlation analysis was used to calculate the correlation between drug IC_50_ values and Riskscore, and the correlation between the two metrics was characterized as significant at *p* < 0.05 and |*c* *o* *r*| > 0.3.

### 2.7. ScRNA‐Seq Analysis

GSE273274 was followed‐up in this study. A minimum of three cells were set up to express each gene in a minimum of 200 genes per cell by the Seurat package [28], retaining the proportion of mitochondrial genes < 15% and the number of genes > 200, and the total number of transcripts ≤ 100,000. Normalization was performed with the SCTransform function, and principal component analysis was performed with the RunPCA function, followed by the use of the harmony package to remove batch effects in different samples. The first 20 principal components were selected for UMAP downscaling. Cell subpopulations were clustered using the FindNeighbors and FindClusters functions, and each cell type was annotated using the marker genes provided by the CellMarker 2.0 database (http://117.50.127.228/CellMarker/).

### 2.8. Spatial Transcriptome Analysis

In this study, spatial transcriptome data of GBM (GSE273275) were read, and SpatialFeaturePlot was used to show the expression of target genes within GBM tissues. FindTransferAnchors and TransferData functions were used to calculate the proportion of immune cell subpopulations within each spot.

### 2.9. Cell Culture and Transfection

The human brain glial cell line HEB (Code No. IM‐H209), human glioma cell line U251 (Code No. IM‐H328), and human GBM astrocytoma cell line U87 (Code No. IM‐H211) used in this study were obtained from IMMOCELL (Xiamen, China, http://www.immocell.com/). Cells were cultured in DMEM supplemented with 10% fetal bovine serum and 1% penicillin–streptomycin at 37°C in a humidified incubator with 5% CO_2_. STR identification has been carried out on the cells, and the outcome of mycoplasma detection shows negativity. To silence *IGFBP6*, transfection of glioma cell lines U251 and U87 with si‐*IGFBP6* (small interfering RNA [siRNA] Sequence #1: 5 ^′^‐GTCTCCAGATGGCAATGGA‐3 ^′^ and siRNA Sequence #2: 5 ^′^‐GAGAATCCTAAGGAGAGTAAACC‐3 ^′^) was performed using the Lipofectamine 2000 Transfection Reagent Kit (11668027, Invitrogen, Carlsbad, California, United States).

### 2.10. RT‐qPCR

The TriZol reagent (15596026, Invitrogen, United States) was used to extract total RNA in cells. The first‐strand cDNA synthesis kit (R211‐01, Vazyme, China) was used to reversely transcribe the RNA into cDNA. RT‐qPCR was performed using the SYBR Green qPCR mixture kit. The relative levels of gene expression were calculated using the 2^−*Δ* *Δ* *C* *t*^ method. The primers used in this study are as follows:

*IGFBP6* forward: CACAGGATGTGAACCGCAGAGA

*IGFBP6* reverse: CACTGAGTCCAGATGTCTACGG

*CTSD* forward: GCAAACTGCTGGACATCGCTTG

*CTSD* reverse: GCCATAGTGGATGTCAAACGAGG

*SPAG4* forward: CTCGTGTTCCAGAGGCTGAATG

*SPAG4* reverse: GCGATTCCAGAAGTAGGCAGTG

*ZNF560* forward: GAAGAGTGGACTTTACTGGACCC

*ZNF560* reverse: CTCTTGGCAGTGTGCTCAACTC

*COL22A1* forward: AAGGTGAACTGGGACTTCCAGG

*COL22A1* reverse: CATCCGTGGATGTGGTGTGAAC

*HOXC13* forward: CAGTCAGGTGTACTGCTCCAAG

*HOXC13* reverse: AGCTGCACCTTAGTGTAGGGCA

*GAPDH* forward: GTCTCCTCTGACTTCAACAGCG

*GAPDH* reverse: ACCACCCTGTTGCTGTAGCCAA

### 2.11. Cell Counting Kit‐8 (CCK‐8) Assay

The cell suspension collected in this study was seeded in a 96‐well plate at a density of 5 × 10^3^ cells per well, with 100 *μ*L per well. CCK‐8 reagent was then added, and the plate was incubated for 30 min. Subsequently, the absorbance of each cell culture system at 450 nm was measured using a microplate reader (Bio‐Rad).

### 2.12. Scratch Healing Assay

Cells were seeded into six‐well plates with 2 mL of complete medium per well and cultured at 37°C in a 5% CO_2_ incubator until a confluent monolayer was formed. A sterile pipette tip was used to create uniform straight scratches across the cell monolayer. The wells were gently washed twice with phosphate‐buffered saline (PBS) to remove detached cell debris, followed by the addition of fresh serum‐free medium. Images of the scratched areas were captured under a microscope immediately (0 h) and 48 h after scratching. The wound healing rate was calculated as follows:

initial width of the scratch−current width of the scratchinitial width of the scratch×100%.

### 2.13. Transwell Analysis

The transfected cells were seeded into the upper chamber of a Transwell device (Corning, United States), then the lower chamber was supplemented with serum‐containing medium. After incubating at 37°C for 48 h, any noninvasive cells were removed from the upper chamber. The cells invaded to the lower chamber were fixed in 4% paraformaldehyde and stained with crystal violet for 10 min. Invaded cells were quantified with an inverted microscope in at least five random fields of view [29].

### 2.14. Statistical Analysis

All statistical analyses were performed using R software. The Log‐rank test was used to compare Kaplan–Meier survival curves between high‐ and low‐risk groups. Immune infiltration comparisons between groups were analyzed using the Wilcoxon rank‐sum test. For *in vitro* cell experiments, GraphPad Prism software was used for experimental data analysis. Data are presented as mean ± standard deviation (SD). Two‐tailed unpaired t‐test, ordinary one‐way ANOVA, and two‐way ANOVA were used as appropriate. A *p* value of < 0.05 was considered statistically significant.

## 3. Results

### 3.1. Identification of Molecular Typing Based on Association With UPSGs in GBM

**Figure 1:**
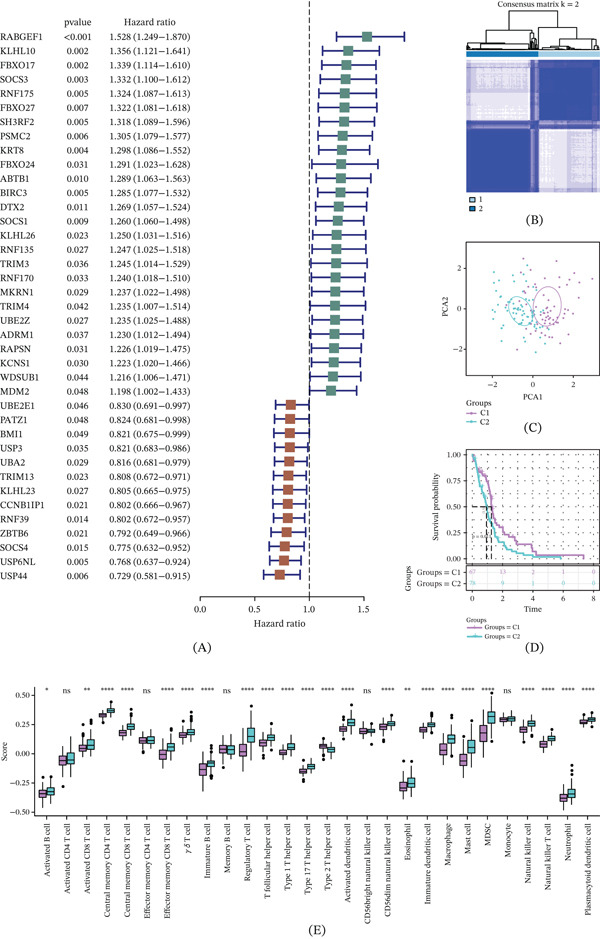
Consistent clustering of GBM. (A) Univariate Cox analysis of UPSGs in TCGA‐GBM. (B) Heatmap of consistency clustering for the TCGA‐GBM dataset. (C) PCA analysis of TCGA‐GBM subtypes. (D) K‐M curves of two molecular subtypes. (E) Comparison of immune cell scores between two molecular subtypes in TCGA‐GBM.

In this study, UPSGs significantly associated with GBM prognosis were clarified by univariate Cox regression analysis, which included 39 genes in total (Figure 1A). By consistency clustering, GBM patients were classified by the expressions of 39 genes, and it was clarified that the number of clusters was 2 with stable clustering results, and finally, two molecular subtypes were obtained in this study (Figure 1B–C). Prognostic analysis of the two subtypes showed notable prognostic survival differences between the subtypes; specifically, the prognosis of C1 subtype was significantly better than that of C2 subtype (Figure 1D). Immune infiltration score was notably distinct between C1 and C2 subtypes, which were significantly enriched to different immune cells and stromal cells (Figure 1E).

### 3.2. Screening of UPS‐Related DEGs in GBM

**Figure 2:**
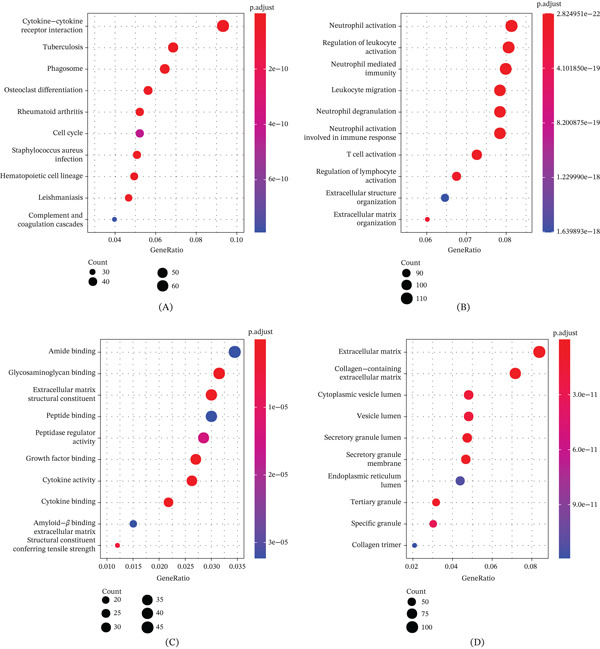
Functional enrichment analysis. (A) Bubble plots of KEGG enrichment analysis of UPS‐associated DEGs. (B–D) Bubble plots of GO‐BP, GO‐MF, and GO‐CC enrichment analyses of UPS‐associated DEGs.

In this study, differential expression analysis of GBM of C1 and C2 subtypes was conducted to filter UPS‐associated DEGs. Subsequently, GO and KEGG enrichment analysis of these DEGs was applied to investigate their involved biological functions and pathways, and it was found that these genes were mainly enriched in the areas of tuberculosis, neutrophil activation, regulation of leukocyte activation, cytokine–cytokine receptor interaction, glycosaminoglycan binding, extracellular matrix, collagen‐containing extracellular matrix, amide binding, collagen‐containing extracellular matrix, and other related functions (Figure 2A–D).

### 3.3. Prognostic Risk Assessment Model for GBM

**Figure 3:**
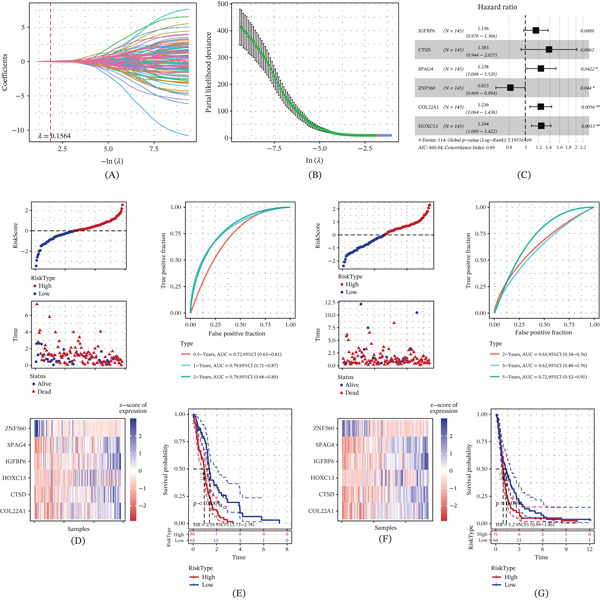
GBM prognostic risk model construction and assessment. (A–B) Distributions of gene coefficients generated with log (*λ*) in the LASSO model and LASSO coefficient profiles. (C) Forest plots of GBM prognosis‐related genes. (D) Expression of Riskscore, survival time versus survival status, and signatures in the TCGA‐GBM. (E) Prognostic model for assessing the TCGA‐GBM prognostic ROC curve and survival curve. (F) Expression of Riskscore, survival time versus survival status, and signatures in mRNAseq_325. (G) Prognostic model used to assess the ROC curve and survival curve of mRNAseq_325 prognosis.

In this study, univariate Cox regression and LASSO regression analyses were conducted on UPS‐related DEGs, 10‐fold cross‐validation was employed to analyze the confidence intervals at different *λ*, revealing that the model reached optimality at lambda = 0.1564 (Figure 3A–B). Through further stepwise regression analysis, six genes were screened as signatures significantly affecting the prognosis of GBM in this study, and used to construct a prognostic model: Riskscore = 0.145 * *IGFBP6* + 0.324 * *CTSD* + 0.213 * *SPAG4* ‐ 0.204 * *ZNF560* + 0.212 * *COL22A1* + 0.219 * *HOXC13* (Figure 3C). Analysis of the TCGA‐GBM training set showed that the prognosis of the GBM samples in the high‐Riskscore group was notably worse than that of the GBM samples in the low‐Riskscore group, and the ROC curves proved that the model was accurate in assessing the prognostic outcome of the patients, with the AUC values of 0.6 or above (Figure 3D–E). The analysis results in the validation set mRNAseq_325 also confirmed the above conclusion that the prognosis of mRNAseq_325 samples in the high‐Riskscore group was significantly worse than that of the low‐Riskscore group, and the model showed a high accuracy in prognostic evaluation, with AUC values of the ROC curves all above 0.6 (Figure 3F–G).

### 3.4. Differential Functional Enrichment of GBM in High‐ and Low‐Riskscore Groups

**Figure 4:**
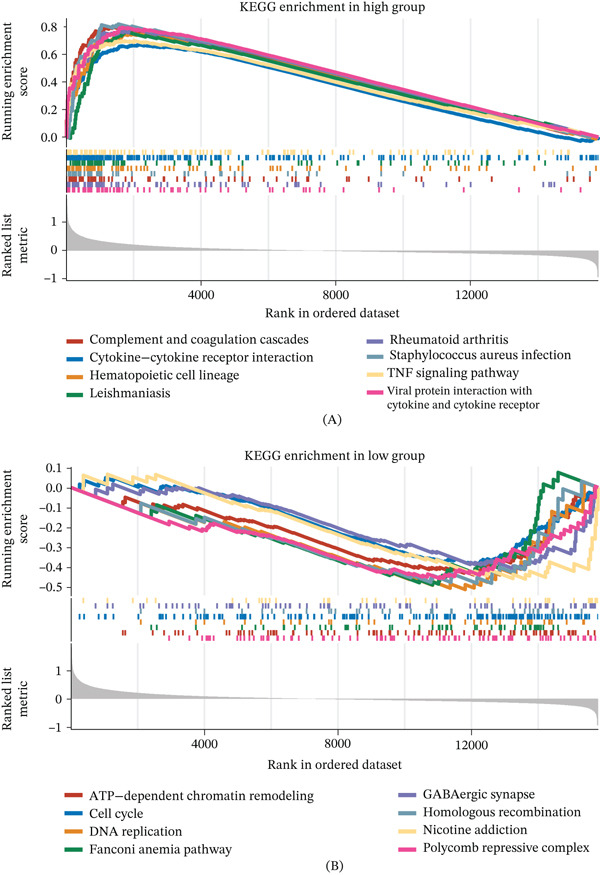
GSEA analysis of TCGA‐GBM high‐ and low‐Riskscore groups. (A) GSEA analysis of TCGA‐GBM high‐Riskscore group. (B) GSEA analysis of TCGA‐GBM low‐Riskscore group.

To investigate the biological pathways and molecular mechanisms associated with GBM prognosis, we performed KEGG enrichment analysis of DEGs between the high‐ and low‐Riskscore groups in the TCGA‐GBM cohort. In the high‐Riskscore group, pathways such as complement and coagulation cascades, viral protein interaction with cytokine and cytokine receptor, cytokine–cytokine receptor interaction, and the TNF signaling pathway were significantly activated. These pathways were primarily related to inflammatory and immune responses (Figure 4A). In the low‐Riskscore group, the significantly enriched pathways included cell cycle, DNA replication, GABAergic synapse, and other pathways related to gene stability, neural signaling, and metabolic regulation (Figure 4B).

### 3.5. Correlation of Riskscore With Immune Microenvironment and Chemotherapeutic Drug Sensitivity in GBM

**Figure 5:**
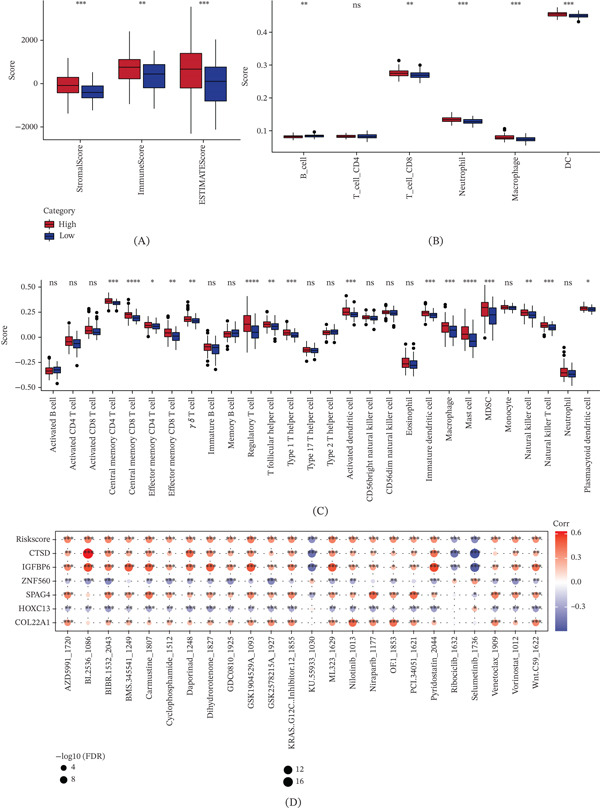
Immunoinfiltration analysis and drug sensitivity assessment of GBM. (A) ESTIMATE analysis, (B) TIMER analysis, and (C) ssGSEA analysis of TCGA‐GBM high‐ and low‐Riskscore groups. (D) Correlation analysis of expression of UPS‐related gene in TCGA‐GBM with drug IC_50_.

ImmuneScore was computed by the ESTIMATE algorithm, and it was observed that the high‐Riskscore group had significantly higher ImmuneScore than the low‐Riskscore group (Figure 5A). TIMER and ssGSEA analyses showed that most of the immune cells were significantly enriched differently in the high‐ and low‐Riskscore groups, including CD8+ T cells and macrophage (Figure 5B–C). To screen potential therapeutic drugs for GBM, the present study further explored the relationship between prognostic modeling and GBM drug sensitivity and found that Riskscore was closely linked to 24 drugs (Figure 5D).

### 3.6. Single‐Cell Mapping of GBM

**Figure 6:**
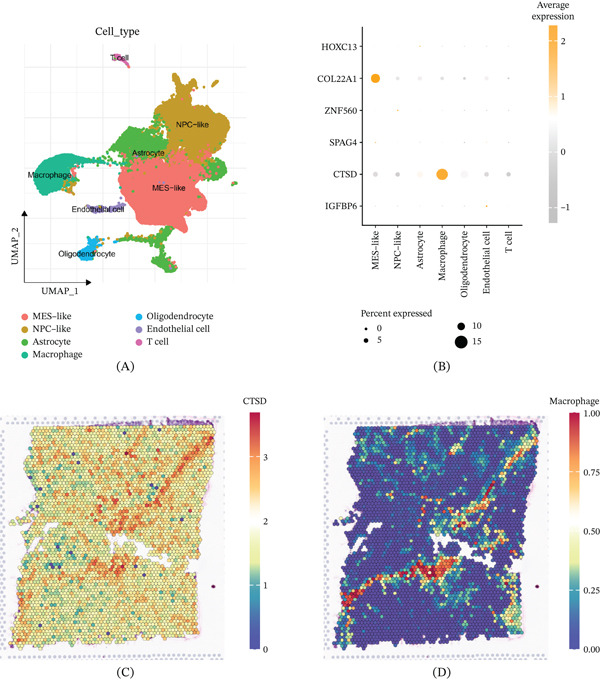
Single‐cell analysis of GBM and spatial transcriptome analysis. (A) UMAP for clustering of each cell subpopulation of GBM. (B) Specific highly expressed genes in different cell subpopulations of GBM. (C) Expression of CTSD in GBM tissues. (D) Macrophage occupancy within GBM tissues.

In order to further explore the cell types involved in the development of GBM, we downloaded three GBM center samples from GSE273274, and after cell filtering, normalization, downgrading, and clustering, 34,403 cells remained, containing 10 major cell subpopulations (Figure S1). In this study, the marker genes were reclassified into seven cellular subpopulations based on their expression in the clusters, including MES‐like, NPC‐like, astrocyte, macrophage, oligodendrocyte, endothelial cell, and T cell (Figure 6A). Among them, *COL22A1* was highly expressed in MES‐like cells, and *CTSD* was highly expressed in macrophages (Figure 6B). To further validate the specific expression of *CTSD* in macrophages, we used spatial transcriptome data for validation and found that *CTSD* was significantly highly expressed in macrophage‐enriched regions, consistent with the results of single‐cell data (Figure 6C–D).

### 3.7. Cellular Role of IGFBP6 in Regulating GBM

**Figure 7:**
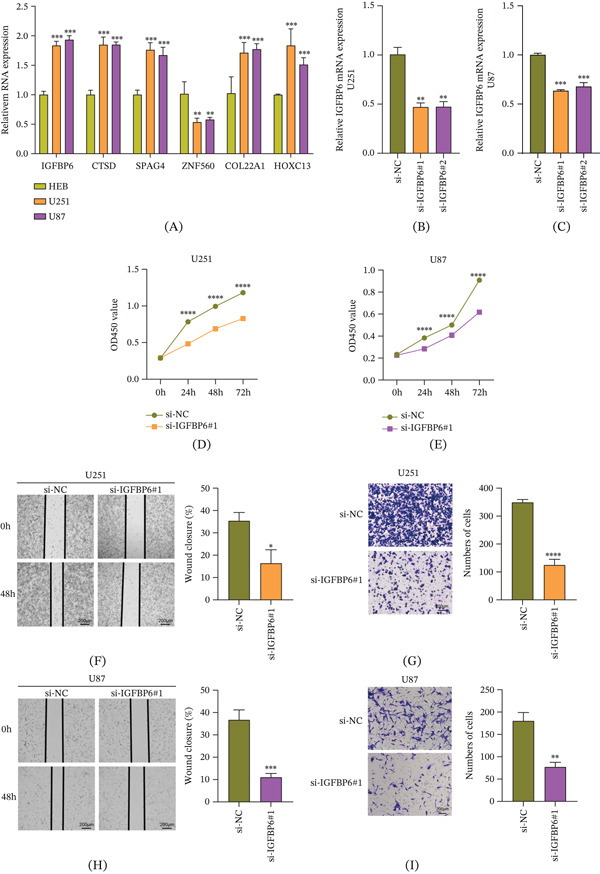
Cell‐level regulatory effects of UPS‐related key genes in GBM. (A) Relative expression levels of six genes associated with GBM progression and the ubiquitin‐proteasome system in HEB, U251, and U87 cell lines. (B) RT‐qPCR analysis of IGFBP6 expression in U251/si‐IGFBP6 cells. (C) RT‐qPCR analysis of IGFBP6 expression in U87/si‐IGFBP6 cells. (D) Cell proliferation of U251/si‐IGFBP6 cells assessed by CCK‐8 assay. (E) Cell proliferation of U87/si‐IGFBP6 cells assessed by CCK‐8 assay. (F) Cell migration of U251/si‐IGFBP6 cells measured by wound healing assay. (G) Cell invasion of U251/si‐IGFBP6 cells assessed by Transwell assay. (H) Cell migration of U87/si‐IGFBP6 cells assessed by wound healing assay. (I) Cell invasion of U87/si‐IGFBP6 cells measured by Transwell assay. ∗*p* < 0.05, ∗∗*p* < 0.01, ∗∗∗*p* < 0.001, and ∗∗∗∗*p* < 0.0001.

In this study, RT‐qPCR was conducted to measure the relative expressions of genes associated in the UPS in HEB, U251, and U87 cell lines, revealing that IGFBP6 was relatively highly expressed in U251 and U87 cells (Figure 7A). IGFBP6‐silenced U251 and U87 cell lines were subsequently constructed (Figure 7B–C). CCK‐8 assays showed that silencing IGFBP6 led to reduced proliferation in both U251 and U87 cells (Figure 7D–E). Furthermore, wound healing and Transwell assays demonstrated decreased migration and invasion capabilities in the IGFBP6‐silenced U251 and U87 cells (Figure 7F–I).

## 4. Discussion

The UPS is one of the major protein degradation mechanisms in cancer cell stress response, which includes ubiquitin‐protein ligases, ubiquitin‐binding enzymes, and deubiquitinating enzymes [30]. For GBM cells, cancer cells in the hypoxic region within the tumor, however, show a strong survival and reproduction ability, which is closely related to the degradation and recycling mechanism of UPS [31, 32]. In this study, we mined key genes in GBM that were closely linked to both disease progression and UPS based on UPS characteristics, and constructed a prognostic risk assessment model for GBM. Meanwhile, this study deeply elucidated the reliability of the model in predicting the diversity of the immune microenvironment of GBM and potential therapeutic drugs for GBM.

In this study, six genes were mined for constructing a GBM prognostic model based on UPS features, including *IGFBP6*, *CTSD*, *SPAG4*, *ZNF560*, *COL22A1*, and *HOXC13*. *IGFBP6* is usually significantly upregulated in GBM, contributes to cancer progression through the activation of cancer immune escape, and is tightly correlated with chemotherapy resistance in GBM [33]. Studies have shown that *IGFBP6* expression regulates microglia polarization and remodels the tumor microenvironment in GBM [34]. The *CTSD* is a gene intimately implicated in the clinical malignant development and prognostic outcomes of GBM, and studies have demonstrated that knockdown of *CTSD* by siRNA or its inhibitor, pepsin inhibitor A, increases GBM radio‐sensitivity [35]. Thus, *CTSD* is expected to serve as a potential target for GBM, which is also consistent with our findings revealing it as a prognostic risk factor for GBM. Similarly, *SPAG4*, a pro‐oncogene in GBM, is upstream regulated by the endoplasmic reticulum transmembrane protein‐encoding gene *ERN1*, and the expression of *SPAG4* reduces the sensitivity of *ERN1* knockdown GBM to a hypoxic environment [36]. *ZNF560* belongs to the zinc‐finger protein ZNF family, whereas the related study showed that the *ZNF554*, which also belongs to the ZNF family, the downregulated expression was associated with activation of pathways related to glioma progression and shortened patient survival, that is, the gene inhibited malignant progression of glioma [37]. This is supportive of the conclusion that this study reveals the existence of *ZNF560* as a prognostic protective factor for GBM. *COL22A1* is a poor prognostic factor for GBM, as demonstrated in a related study based on cellular experiments that downregulation of *COL22A1* reduces the growth and proliferation of GBM cells [38]. *HOXC13* and its family members *HOXC6*, *HOXC8*, and *HOXC10* were all shown to be predictors of poor GBM prognosis and significantly associated with immune cell infiltration in the GBM immune microenvironment [39]. Thus, the UPS‐related genes identified in this study, all of which are associated with GBM progression, may serve as valuable biomarkers for prognostic assessment of GBM.

To further clarify the cellular origin and potential mechanisms of action of the UPS‐related genes identified in GBM, we conducted a detailed analysis of GBM cell subpopulations using single‐cell transcriptomic data. In this study, GBM was categorized into seven cellular subpopulations according to the expressions of marker genes in GBM, among which *CTSD* was significantly highly expressed in macrophages and confirmed by spatial transcriptome analysis. Meanwhile, immune infiltration analysis revealed that macrophages were significantly infiltrated in the high Riskscore group. The regulation of macrophage infiltration level and activity by *CTSD* provides insights into the present study, which may explain the development of GBM, as a bioinformatics study clarified that *CTSD* acts as a pivotal gene associated with the progression of M2 macrophages and that metrics associated with it can be used to assess cancer patients′ sensitivity to immunotherapy [40]. In addition, *CTSD*, which is upregulated in macrophages, was present as a UPS‐associated signature gene in this study, and related studies have clarified that knockdown of *CTSD* induced an increase in ubiquitin‐associated and autophagy‐associated proteins and activated microglia and astrocytes, which induced significant expression of inflammatory markers in the cells [41]. Thus, it cannot be ruled out that *CTSD* may affect macrophage activity, which in turn promotes the release of their inflammatory factors and consequently affects GBM development.

This study had several limitations. First, the sample sizes used for model development and validation were relatively modest. In the future, larger multicenter independent cohorts, such as additional GBM datasets from GEO and our own clinical samples, should be included to externally validate the model and improve its generalizability. Second, the single‐cell analysis was based on only three GBM samples, which may limit the representativeness of cell subpopulations. Collecting more single‐cell datasets will be necessary to fully map the expression patterns of UPS‐related signature genes in the GBM microenvironment. In addition, this study lacks tissue‐level evidence to demonstrate direct interactions between UPS‐related signature genes, such as *CTSD*, and macrophages. Future investigations will employ coculture systems, immunohistochemistry, and spatial proximity analyses to verify these regulatory relationships. Lastly, the prediction of immunotherapy sensitivity based on the model has not yet been validated in clinical cohorts. Therefore, integrating data from immunotherapy trials will be essential to assess the practical value of the model in identifying patients who are most likely to benefit from immune checkpoint blockade.

## 5. Conclusion

In summary, this work builds a UPS‐related six‐gene signature that predicts survival in GBM. Higher risk scores come with more immune cell infiltration, especially macrophages and CD8^+^ T cells, and are linked to different drug sensitivity profiles. Single‐cell and spatial data show that certain key genes, such as *CTSD* and *COL22A1*, are enriched in specific cell populations. Functional experiments also confirm that IGFBP6 drives GBM cell proliferation, migration, and invasion. These results point to new biomarkers and potential treatment targets for GBM.

**Nomenclature**
AUC: area under the curve
CGGA: Chinese Glioma Genome Atlas
DEGs: differentially expressed genes
GBM: glioblastoma
GEO: Gene Expression Omnibus
GO: Gene Ontology
IC_50_
: inhibitory concentration in half
KEGG: Kyoto Encyclopedia of Genes and Genomes
KM: Kaplan–Meier
PCA: principal component analysis
ROC: receiver operating characteristic curve
ssGSEA: single‐sample gene set enrichment analysis
scRNA‐Seq: single cell RNA sequencing
UPS: ubiquitin‐proteasome system

## Author Contributions

All authors contributed to this present work: Hai Jin and Da Huo conducted research design, data analysis, and draft writing. Zheng Yu assisted in basic experimental verification and data collection. Wanting Xie and Yue Feng assisted in data analysis and guided the revision of articles. Da Huo and Yue Feng contributed equally to this work.

## Funding

This study was supported by Cultivation Plan for Young Talents in Military Medical Science and Technology (2026LBQNPY023).

## Disclosure

All authors read and approved the manuscript

## Ethics Statement

Ethical approval was not required for this study because it is not involved in any human experiments.

## Consent

The authors have nothing to report.

## Conflicts of Interest

The authors declare no conflicts of interest.

## Supporting information


**Supporting Information** Additional supporting information can be found online in the Supporting Information section. Figure S1: Single‐cell subpopulation clustering in GBM. (A) UMAP plot showing cell distribution across three integrated samples (GSM8425587, GSM8425589, and GSM8425591). (B) Violin plots of cross‐sample quality control metrics, including number of features detected per cell (nFeature_RNA), total RNA count per cell (nCount_RNA), and percentage of mitochondrial genes (percent.mt). (C) UMAP plot of cell clusters identified by Seurat analysis. (D) Scatterplot of expression patterns for representative marker genes in major cell types. The color scale indicates average expression levels, whereas dot size represents the proportion of cells expressing the marker gene.

## Data Availability

The datasets generated and/or analyzed during the current study are available in the [GSE273274] repository, [https://www.ncbi.nlm.nih.gov/geo/query/acc.cgi?acc= GSE273274], and the [GSE273275] repository, [https://www.ncbi.nlm.nih.gov/geo/query/acc.cgi?acc= GSE273275].
