# Supplementary figures and images for: Ubiquitin‐Proteasome System–Related Prognostic Model and Immune Landscape in Glioblastoma
Source: Int J Genomics. 2026 Jul 22;2026:2045937. doi: 10.1155/ijog/2045937 (PMC13390022; doi:10.1155/ijog/2045937)

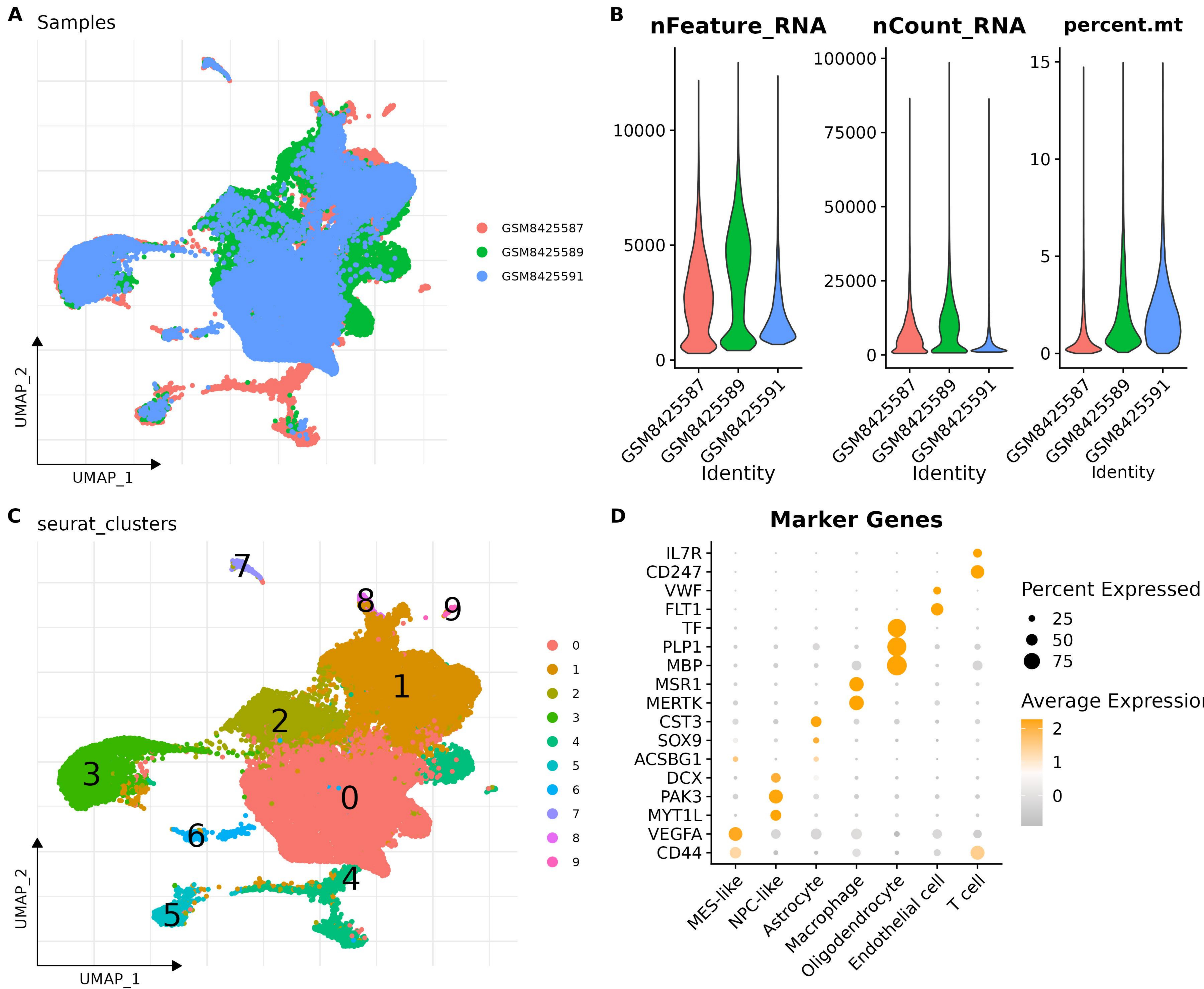

Supplement: Supplementary file 1 — Supporting Information Additional supporting information can be found online in the Supporting Information section. Figure S1: Single‐cell subpopulation clustering in GBM. (A) UMAP plot showing cell distribution across three integrated samples (GSM8425587, GSM8425589, and GSM8425591). (B) Violin plots of cross‐sample quality control metrics, including number of features detected per cell (nFeature_RNA), total RNA count per cell (nCount_RNA), and percentage of mitochondrial genes (percent.mt). (C) UMAP plot of cell clusters identified by Seurat analysis. (D) Scatterplot of expression patterns for representative marker genes in major cell types. The color scale indicates average expression levels, whereas dot size represents the proportion of cells expressing the marker gene. [file IJOG-2026-2045937-s001.pdf]
